# Nationwide Short-Term and Midterm Clinical Outcome Comparison of Hybrid Coronary Revascularization Versus Off-Pump Coronary Artery Bypass Surgery

**DOI:** 10.1177/15569845261437076

**Published:** 2026-04-23

**Authors:** Monica Gianoli, Anne R. de Jong, Ferenc van der Hulst, Maaike M. Roefs, Sandeep Singh, Patrique Segers, Pim van der Harst, Willem J.L. Suyker, Kirolos A. Jacob

**Affiliations:** 1 Heart and Lungs Division, Cardiothoracic Department, University Medical Center Utrecht, The Netherlands; 2 Netherlands Heart Registration, Utrecht, The Netherlands; 3 Cardiothoracic Department, Isala Clinics, Zwolle, The Netherlands; 4 Cardiothoracic Department, Maastricht University Medical Center, The Netherlands; 5 Heart and Lungs Division, Cardiology Department, University Medical Center Utrecht, The Netherlands

**Keywords:** hybrid revascularization, off-pump coronary artery bypass, minimal invasive cardiac surgery, robotic-assisted MIDCAB

## Abstract

**Objective:**

Hybrid coronary revascularization (HCR) has emerged as a minimally invasive alternative to conventional coronary artery bypass techniques for patients with multivessel coronary artery disease. This study aimed to compare the clinical outcomes of HCR versus off-pump coronary artery bypass (OPCAB) in patients with multivessel disease in The Netherlands.

**Methods:**

This study analyzed 117 HCR patients and 313 OPCAB patients after propensity score matching. The primary outcome was the incidence of major adverse cardiac and cerebrovascular events (MACCE) at 30 days after the procedure. Secondary outcomes included mortality, myocardial infarction, repeat revascularization, and ischemic cerebrovascular accidents. Midterm survival and repeat target vessel revascularization (TVR) were evaluated over a median follow-up of 36 months.

**Results:**

At 30 days, MACCE rates were similar between the HCR group (5.1%) and the OPCAB group (4.1%; *P* = 0.62). Secondary outcomes were also comparable between the groups. The incidence of myocardial infarction in the HCR group was numerically higher (3.4% vs 1.6%), but this difference was not statistically significant (*P* = 0.17). Midterm survival rates showed no significant difference between the groups, although numerically the HCR showed a higher rate of TVR (5.1% vs 4.1%, *P* = 0.62). No significant differences were found in blood transfusion requirements or postoperative hospital stay duration.

**Conclusions:**

HCR demonstrated comparable short-term and midterm outcomes to OPCAB, suggesting it is a viable alternative for multivessel coronary artery disease treatment. However, further research is necessary to assess long-term effectiveness and to identify specific patient populations who may benefit most from HCR.

## Graphical abstract

**Visual abstract:**
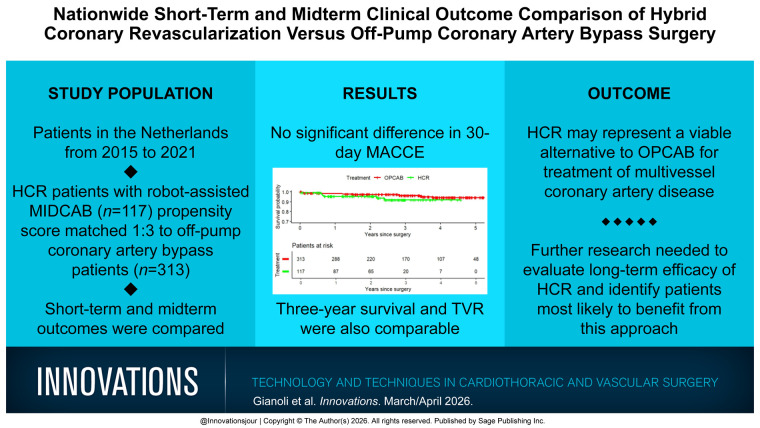

**Central Message**

HCR demonstrated similar short-term and midterm outcomes compared with OPCAB in patients with multivessel coronary artery disease. These findings support HCR as a safe and minimally invasive alternative, although further studies are needed to evaluate long-term outcomes and define ideal patient selection.

## Introduction

Off-pump coronary artery revascularization (OPCAB) offers benefits compared with traditional coronary artery bypass grafting (CABG) by avoiding the trauma of cardiopulmonary bypass and minimizing aortic manipulation.^1,2^ However, concerns persist regarding the completeness and quality of coronary revascularization, potentially leading to an increased need for repeat revascularization and late mortality.^3^ Technical challenges in achieving an optimal anastomosis become more evident when dealing with the lateral and inferior heart walls, which are more difficult to reach in a beating heart setting.^4,5^ In contrast, anterior wall anastomoses, specifically for the left anterior descending coronary artery (LAD), appear to be more accurate, comparable with CABG, ensuring the patient’s survival benefit from the left internal mammary artery (LIMA) to the LAD.^4–7^ From this perspective, hybrid coronary revascularization (HCR) might represent an advancement of OPCAB toward an even more minimally invasive approach. Essentially, it combines the off-pump LIMA-LAD grafting through a sternal-sparing small anterolateral thoracotomy, namely, minimally invasive direct coronary artery bypass (MIDCAB), with percutaneous coronary intervention (PCI) for non-LAD coronaries.

Currently, in The Netherlands, OPCAB is performed in only a small percentage of surgical revascularizations, and its use has declined over the past decade.^8,9^ However, the hybrid approach gained increasing interest and seems to hold promise in this field. This shift in surgical strategy toward more minimally invasive approaches lacks strong supporting evidence, primarily due to the limited availability of large observational or randomized studies.

In The Netherlands, HCR is currently performed in combination with robot-assisted MIDCAB (RA-MIDCAB) at 3 specialized cardiac centers. Patient data from these procedures are consolidated into the national Netherlands Heart Registration (NHR), which is updated annually to provide a comprehensive, nationwide registry. The objective of this study is to evaluate whether HCR in The Netherlands yields outcomes comparable with those of OPCAB.

## Methods

### Patient Population

We conducted a retrospective analysis comparing patients who received HCR with those who underwent OPCAB. Our study included 117 patients who underwent HCR at 3 cardiac centers in The Netherlands (University Medical Center Utrecht [UMCU], Maastricht University Medical Center, and Isala Clinics Zwolle) from January 2015 to December 2021. Patients had multivessel coronary artery disease and received RA-MIDCAB with LIMA-LAD conduit, along with PCI for non-LAD vessels. Eligibility required documentation of the treatment plan during heart team meetings. All patients underwent a two-staged HCR (RA-MIDCAB followed by PCI) or reversed two-staged HCR (PCI first, then RA-MIDCAB for the LAD lesion), with the interval between the 2 procedures not exceeding 3 months. Adults (≥18 years) with proximal or chronically occluded LAD lesions and at least 1 left circumflex or right coronary artery lesion treatable with PCI were included. Patients in whom the heart team identified a contraindication for RA-MIDCAB (e.g., severe pulmonary disease) or a contraindication for PCI (e.g., inability to tolerate dual antiplatelet therapy) were not scheduled for HCR and therefore not part of this cohort. No additional exclusions were applied retrospectively by the authors.

The study included 439 patients considered for OPCAB treatment at UMCU during the same period. Inclusion criteria were LAD disease with at least 1 additional coronary lesion. Exclusions included single-vessel OPCAB, reoperations, traditional CABG, and concurrent procedures. Preoperative mechanical circulatory or ventilation support use was excluded for both groups.

The study was approved by the ethical board (METC 21-623/C), and written consent was obtained for RA-MIDCAB patients. Informed consent for OPCAB was waived.

### Data Collection

This was an observational, retrospective, cohort study of prospectively collected data registered within the NHR.^8–10^ Additional HCR data were collected by participating centers. Study approval was obtained from the institutional review board MEC-U (W19.270), and the study was conducted in accordance with the Declaration of Helsinki. A waiver for informed consent for analysis with the data of the NHR data registry was obtained. OPCAB data were extracted from hospital electronic records and manually verified. Missing data were manually integrated.

### Outcomes

The primary outcome was the incidence of major adverse cardiac and cerebrovascular events (MACCE) within a 30-day follow-up period. MACCE was defined as a composite outcome comprising all-cause mortality, myocardial infarction (MI), cerebrovascular accident (CVA), new-onset renal failure necessitating dialysis, as well as instances of surgical and/or percutaneous repeat revascularization within 30 days postoperatively. MI was defined according to the Fourth Universal Definition of Myocardial Infarction.^11^ Other complications were classified based on the European System for Cardiac Operative Risk Evaluation (EuroSCORE).^12^ Only first events were considered within the MACCE criteria.

Secondary outcomes included individual MACCE components, all-cause mortality, and target vessel repeat revascularization (TVR) during midterm follow-up. Mortality was evaluated within 30 days and beyond, with events cross-referenced against the Dutch Nationwide Personal Records database for confirmation. Cardiac origin was presumed for all deaths unless a clear noncardiac cause was identified. When the cause of death was unclear, efforts were made to contact patients’ general practitioners for additional information. TVR was defined as subsequent revascularization of the target vessels from the initial procedure, considering only the first event. Given the variability in inclusion dates within the HCR cohort across the participating centers, where RA-MIDCAB was introduced between 2015 and 2018, a common follow-up duration approach for midterm outcomes was adopted. The description of the surgical techniques and PCI are provided in the Supplemental Material (Appendix 2).

### Statistical Analysis

Continuous variables are reported as means with standard deviations or medians with interquartile ranges (IQRs) for non-normal distributions, whereas categorical variables are presented as numbers with percentages. All baseline clinical and surgical variables (Table 1) were considered potential confounders or variables leading to selection bias for either treatment, based on previous literature, and hence were included in the multivariable logistic regression model for propensity score matching (PSM). All variables were force entered in the a priori logistic regression model. This was done to ensure all relevant variables were considered, and this method is suitable given that, based on previous literature, we had strong hypotheses about the relationship between the baseline variables and the treatment arms.^12^ These covariates comprised age, sex, body mass index, diabetes mellitus, peripheral vascular disease (PVD), chronic obstructive pulmonary disease, left ventricular (LV) grade, MI, preoperative CVA, and preoperative serum creatinine. Employing a 1:3 PSM analysis, we computed weighted odds ratios (ORs) and their respective confidence intervals (CIs) through weighted logistic regression. Continuous outcomes were analyzed using weighted linear regression to calculate weighted mean differences.

**Table 1.** Patient Demographics Before and After PSM for Patients Who Underwent HCR Compared With Patients Who Underwent OPCAB Between 2015 and 2021 in The Netherlands.

	Study population before PSM				Study population after PSM			
Patient characteristics	HCR (*n* = 117)	OPCAB (*n* = 439)	*P* value	SMD	HCR (*n* = 117)	OPCAB (*n* = 313)	*P* value	SMD
Age, years	64.3 ± 10.5	66.6 ± 10	0.02	0.227	64.3 ± 10.5	65.3 ± 10	0.22	0.096
Male	95 (81.2)	357 (81.1)	0.93	−0.003	95 (81.2)	258 (82.4)	0.77	−0.033
BMI, kg/m^2^	26.8 ± 3.6	27.0 ± 4.2	0.78	0.049	26.8 ± 3.6	26.9 ± 4	0.75	0.025
LVEF >50%	113 (96.6)	391 (88.9)	0.01	−0.264	113 (96.6)	305 (97.4)	0.63	−0.017
Recent MI <90 days	29 (24.8)	121 (27.5)	0.59	−0.018	29 (24.8)	81 (25.9)	0.80	−0.009
DM	23 (19.7)	111 (25.2)	0.21	−0.045	23 (19.7)	65 (20.8)	0.63	−0.010
CVA	4 (3.4)	16 (3.6)	0.91	−0.121	4 (3.4)	10 (3.2)	0.91	0.082
PVD	8 (6.8)	62 (14.1)	0.03	−1.580	8 (6.8)	29 (9.3)	0.42	−0.097
COPD	10 (8.5)	39 (8.9)	0.91	−0.012	10 (8.5)	28 (8.9)	0.90	−0.010
Creatinine, μmol/L	91.1 ± 23.2	90.0 ± 42.7	0.78	−0.028	91.1 ± 23.2	88.4 ± 32.3	0.43	−0.096
Dialysis	0	2 (0.4)	0.46	−0.707	0	0	—	0

Abbreviations: BMI, body mass index; COPD, chronic obstructive pulmonary disease; CVA, cerebrovascular accident; DM, diabetes mellitus; HCR, hybrid coronary revascularization; LVEF, left ventricular ejection fraction; MI, myocardial infarction; OPCAB, off-pump coronary artery bypass; PSM, propensity score matching; PVD, peripheral vascular disease; SMD, standardized mean difference.

Data are reported as counts (%) or mean ± standard deviation.

In evaluating the ORs or means between the HCR and OPCAB groups, differences were assessed relatively. For adverse events such as mortality, a value less than 1 favored the hybrid group. Statistical significance was considered when the CI did not encompass 1 and the *P* value was less than 0.05. Kaplan–Meier curves were constructed to examine all-cause mortality and freedom from revascularization following the index operation. Statistical analyses were performed using R version 4.3.1 (The R Foundation for Statistical Computing, Vienna, Austria) and IBM SPSS Statistics for Windows, Version 26 (IBM Corp, Armonk, NY, USA). R packages included “MatchIt,” “tableone,” “survival,” “survminer,” “epitools,” “stats,” and “ggplot2.”

## Results

Before PSM, the HCR and OPCAB groups exhibited imbalances in several key variables. Patients undergoing HCR were statistically significantly younger, had better LV function, and had a lower incidence of PVD compared with those undergoing OPCAB. Significant imbalances, indicated by a standardized mean difference (SMD) of >0.25, were observed for preoperative dialysis, LV function, and PVD. Moderate imbalances were detected in age and CVA, favoring the HCR group.

**Fig. 1.**
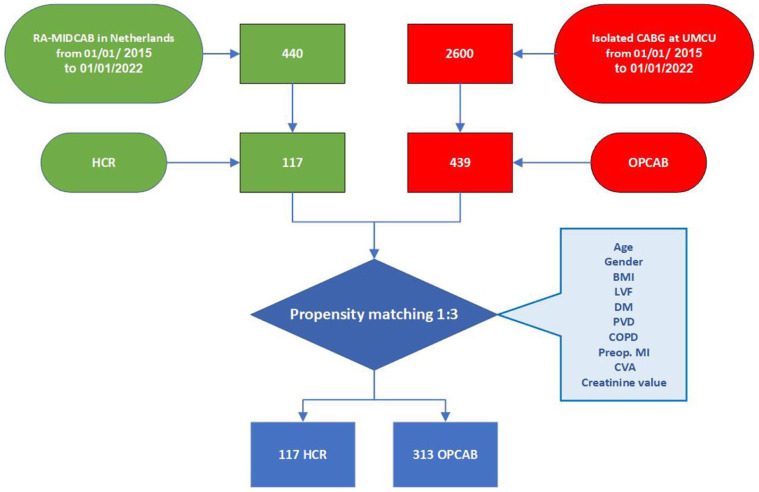
Flow diagram to PSM for patients undergoing HCR and OPCAB. BMI, body mass index; CABG, coronary artery bypass grafting; COPD, chronic obstructive pulmonary disease; CVA, cerebrovascular accident; DM, diabetes mellitus; HCR, hybrid coronary revascularization; LVF, left ventricle ejection fraction; OPCAB, off-pump coronary artery bypass; preop MI, preoperative myocardial infarction; PSM, propensity score matching; PVD, peripheral vascular disease; RA-MIDCAB, robot-assisted minimally invasive direct coronary artery bypass; UMCU, University Medical Center Utrecht.

Following PSM, the analysis included 117 patients who underwent HCR and 313 patients who underwent OPCAB, as depicted in Figure 1. The average age of the patients was 64 ± 10 years in the HCR group and 65 ± 10 years in the OPCAB group. Of the HCR patients, 81% (*n* = 95) were male, whereas in the OPCAB group, 82% (*n* = 258) were male. In both groups, 97% of all patients exhibited good or moderate LV function (113 in the HCR group, 305 in the OPCAB group). None of the variables showed a significant difference after PSM. We estimated propensity scores using logistic regression based on baseline characteristics. Prior to matching, the distribution of scores showed some overlap between the HCR and OPCAB groups, with all patients falling within the region of common support (Supplemental Fig. 1). After matching, covariate balance with substantial overlap was achieved (Supplemental Fig. 2). The substantial reduction in SMD to <0.1 for critical variables such as age, good LV function, and PVD indicates that the matching process successfully mitigated potential confounding effects.

Demographics, cardiovascular risk factors, and comorbidities of the included patients in the 2 groups before and after PSM are listed in Table 1. A higher percentage of patients in the OPCAB group required urgent revascularization, including 161 patients (51.4%) compared with 20 patients (17%) in the HCR group. However, within the HCR group, 61 patients (52%) underwent urgent PCI as part of a reverse two-staged procedure, highlighting an alternative approach for managing urgent cases. Further, in the HCR group, 93 patients (79%) had two-vessel disease, and nearly all patients (91%) received drug-eluting stents. In the OPCAB group, 112 patients (35.8%) had two-vessel disease and 201 (64.2%) had three-vessel disease. The average number of distal anastomoses was 3.6±1.1, corresponding closely with the extent of coronary disease. All patients received complete surgical revascularization, with 100% undergoing LIMA to LAD grafting. Furthermore, 61.6% of OPCAB patients received at least a second arterial anastomosis, primarily involving the LIMA to the diagonal artery, and approximately 20% of patients received a composite Y-graft construction. There were no cases that necessitated the use of the heart–lung machine. A summary of the operative characteristics of patients included in the HCR and OPCAB groups after PSM is provided in Supplemental Table 1.

### Primary Outcome: MACCE

A complete follow-up was conducted, including assessments during hospitalization and at both 30 days postoperative. In the HCR group, 6 patients (5%) experienced a MACCE whereas in the OPCAB group, 13 patients (4.1%) had a MACCE (OR = 1.25, 95% CI: 0.47 to 2.80, *P* = 0.62). Upon analysis of the 30-day outcome, no difference was found between the HCR and OPCAB groups in the composite outcome of MACCE (Table 2).

**Table 2.** Primary Outcome (MACCE) and Secondary Outcomes In-Hospital and at 30-Day Follow-Up Comparing HCR With OPCAB After Propensity Score Matching.

	HCR (*n* = 117)	OPCAB (*n* = 313)	Odds ratio	95% CI	*P* value
MACCE	6 (5.1)	13 (4.1)	1.25	0.47–2.80	0.623
All-cause mortality	1 (0.9)	3 (1.0)	0.89	0.05–4.80	0.913
Cardiac	1 (0.9)	0			
Respiratory	0	2 (0.6)			
Neurologic	0	1 (0.3)			
Perioperative MI	4 (3.4)	5 (1.6)	2.18	0.61–6.13	0.173
RR	5 (4.3)	7 (2.2)	1.70	0.64–4.90	0.188
PCI	3 (2.6)	1 (0.3)			
Surgical revision	2 (1.7)	6 (1.9)			
iCVA	1 (0.9)	4 (1.3)	0.67	0.04–3.43	0.697
New kidney failure requiring dialysis	0	0	—^a^	—^a^	—
Reoperation	4 (3.4)	12 (3.8)	0.89	0.26–2.27	0.825
Bleeding/tamponade	2 (1.7)	5 (1.6)			
Surgical graft revision	2 (1.7)	6 (1.9)			
Others	0	1 (0.3)			
Kidney failure	1 (0.9)	1 (0.3)	—^a^	—^a^	0.977
RBC transfusion	12 (10.3)	38 (12.1)	0.56	0.26–1.07	0.103
ICU length of stay, days	1.1 ± 0.9	1.3 ± 1.8	0.99	0.98–1.01	0.466
Hospital length of stay, days	4.8 ± 3.8	5.5 ± 3.5	0.99	0.99–1.00	0.274

Abbreviations: CI, confidence interval; HCR, hybrid coronary revascularization; ICU, intensive care unit; iCVA, ischemic cerebrovascular accident; MACCE, major adverse cardiac and cerebrovascular events; MI, myocardial infarction; OPCAB, off-pump coronary artery bypass; PCI, percutaneous coronary intervention; RBC, red blood cells; RR, repeat revascularization.

Data are reported as counts (%) or mean ± standard deviation unless otherwise indicated. Mortality is considered as in-hospital plus 30 days.

a Data are not given because the odds ratio and 95% CI were very wide and unstable due to the small number of events. Hence, no comparisons could be made.

### Secondary Outcomes at 30-Day Follow-Up

Within the 30-day outcomes, there were no differences between the HCR and OPCAB groups regarding any of the MACCE individual components. Furthermore, there were no significant differences in the need for blood transfusions or the duration of postoperative hospital stays between the 2 groups (Table 2).

#### Death

In the HCR group, 1 patient (0.9%) died after experiencing a perioperative MI and subsequent heart failure despite surgical revision. In the OPCAB group, 3 patients (1%) died; 2 patients from respiratory failure and 1 patient from neurologic impairment following a postoperative ischemic CVA.

#### Myocardial infarction

In the HCR group, 4 patients (3.4%) experienced a perioperative MI. Two of these patients underwent PCI of the LAD, and 2 patients required reoperation to address an anastomosis issue. No MIs were related to the interval between the 2 procedures. In the OPCAB group, 5 patients (1.6%) experienced a perioperative MI (*P* = 0.17). One of these patients underwent PCI for the LAD and right coronary artery, 3 patients required surgical revisions, and 1 patient received pharmacological treatment.

#### Repeat revascularization

Repeat revascularization was required in 5 HCR patients (4.3%). In addition to the 4 patients who experienced MI, 1 patient underwent PCI with balloon dilatation of the LIMA conduit due to ischemic electrocardiography (ECG) changes. In the OPCAB group, 7 patients (2.2%) required repeat revascularization. Four patients underwent repeat procedures after MI, whereas 3 patients required surgical revision for graft failure confirmed by coronary catheterization following abnormal ECG findings. Two of these cases occurred within hours after the initial surgery, and the third was identified immediately after surgery.

#### Ischemic CVA

One patient (0.9%) developed an ischemic CVA after HCR, whereas 4 patients (1.3%) experienced an ischemic CVA after OPCAB.

#### Reoperations

In the HCR group, 4 patients (3.4%) required reoperation: 2 for graft revision (1 via sternotomy, 1 via minithoracotomy) and 2 for postoperative bleeding. In the OPCAB group, 12 patients (3.8%) required reoperation: 6 for graft revision, 5 for bleeding or tamponade, and 1 for mediastinitis.

### Secondary Outcomes at Median Follow-Up

**Fig. 2.**
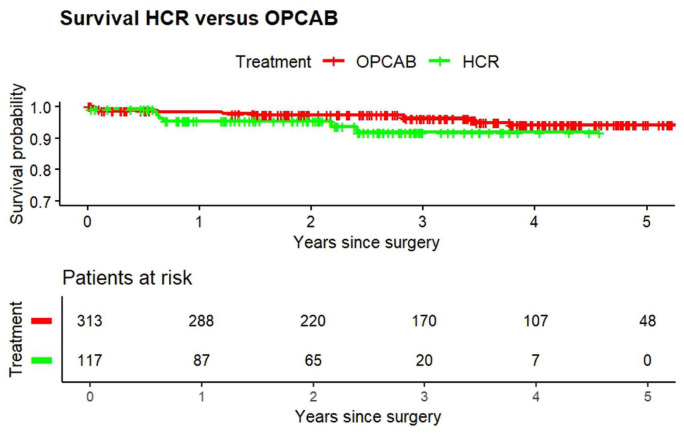
Kaplan–Meier curve representing patient survival after HCR and OPCAB with midterm follow-up of 2,000 days. HCR, hybrid coronary revascularization; OPCAB, off-pump coronary artery bypass.

In the HCR group, at a midterm follow-up of 36.6 (IQR 23.9 to 47.2) months, 8 patients (6.8%) died (Table 3, Fig. 2). Among these, 2 patient deaths were attributed to cardiac causes. One patient has been described before, and 1 patient had an in-hospital unwitnessed cardiac arrest. One patient death was due to an infectious cause, another patient’s cause of death was neurologic, and 3 patients succumbed to oncologic conditions. In the OPCAB group, at a median follow-up of 37.7 (IQR 21 to 53.6) months, 16 patients (5.1%) died. Three of these patients died within the first 30 days postoperatively, as previously described. One patient death was attributed to a cardiac cause, whereas the cause of death for 3 patients remains unknown due to the inability to access their medical records. Among the remaining causes of death, 5 patients succumbed to oncologic conditions, 1 patient to respiratory disease, 1 patient to a neurologic condition, and 2 patients following acute hospital admissions due to abdominal bleeding and renal complications.

**Table 3.** Mortality and RR at Midterm Follow-Up Comparing HCR With OPCAB After Propensity Score Matching.

Outcomes at >30 days of follow-up	HCR (*n* = 117)	OPCAB (*n* = 313)	Odds ratio	95% CI	*P* value
Mortality (excluding first 30 days)	7 (6.0)	13 (4.1)	1.18	0.48–2.51	0.69
Follow-up time, months	36.6 (23.9–47.2)	37.7 (21.0–53.6)			
Alive at follow-up	109 (93.2)	297 (94.9)			
Total mortality (including first 30 days)	8 (6.8)	16 (5.1)	1.63	0.58–2.80	0.43
Cardiac	2 (1.7)	1 (0.3)			
Respiratory	0	3 (1.0)			
Oncologic	3 (2.6)	5 (1.6)			
Neurologic	2 (1.7)	2 (0.6)			
Unknown	0	3 (1.0)			
Others	1 (0.9)	2 (0.6)			
TVR (excluding first 30 days)	6 (5.1)	13 (4.1)	1.25	0.47–2.80	0.62
Repeat PCI	6 (5.1)	10 (3.2)	1.35	0.45–3.27	0.54
Repeat CABG	0	3 (0.9)	—^a^	—^a^	0.98
Total RR + TVR (from initial operation)	11 (9.4)	19 (6.1)	1.61	0.78–3.04	0.17
Follow-up for repeat PCI, months	34.6 (23.0–46.1)	36.5 (18.3–53.0)			
Follow-up for repeat CABG, months	36.4 (23.9–46.8)	37.5 (20.3–53.4)			
Freedom from any revascularization	106 (90.6)	294 (93.9)			

Abbreviations: CABG, coronary artery bypass graft; CI, confidence interval; HCR, hybrid coronary revascularization; OPCAB, off-pump coronary artery bypass; PCI, percutaneous coronary intervention; RR, repeat revascularization; TVR, target vessel revascularization.

Data are reported as counts (%) or median (interquartile range) unless otherwise indicated.

a Data are not given because the odds ratio and 95% CI were very wide and unstable due to the small number of events. Hence, no comparisons could be made.

**Fig. 3.**
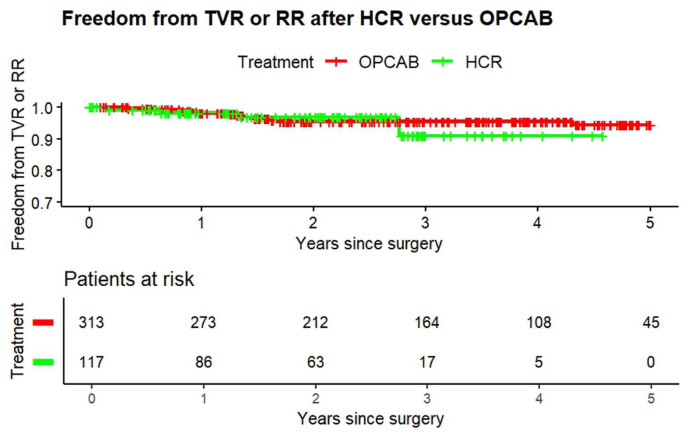
Kaplan–Meier curve representing freedom from RR and TVR after HCR and OPCAB with midterm follow-up of 2,000 days. HCR, hybrid coronary revascularization; OPCAB, off-pump coronary artery bypass; RR, repeat revascularization; TVR, target vessel revascularization.

In the HCR group, at a midterm follow-up of 34.6 (IQR 23 to 46.1) months, 6 patients (5.1%) underwent TVR (Table 3, Fig. 3). Among these, 3 patients received PCI for the LAD, 2 patients underwent repeat PCI for the right coronary artery, and 1 patient underwent PCI for the circumflex coronary artery. In the OPCAB group, 13 patients (4.1%) underwent TVR. Three patients required a repeat CABG at a median follow-up of 37.5 (IQR 20.3 to 53.4) months, and 10 patients underwent repeat PCI at a median follow-up of 36.4 (IQR 18.3 to 53) months. Specifically, 4 patients underwent PCI for the right coronary artery, 3 for the circumflex artery, 2 for the LAD, whereas 1 patient underwent PCI for the vein graft.

## Discussion

In The Netherlands, HCR showed no significant difference in postoperative outcomes compared with OPCAB at both short-term and midterm follow-ups. Within the 30-day postoperative period, there was no significant difference in cumulative MACCE outcomes between the 2 procedures. Over a median follow-up of 3 years, HCR demonstrated comparable outcomes with OPCAB in terms of survival and TVR.

These findings align with the current literature. Recently, Hage et al. reported similar results regarding MACCE and survival. However, their study did show the benefit of HCR over OPCAB in terms of the need for blood transfusion and hospital length of stay.^13^ In our study, we did not find such advantages. Hospital stays were similar between procedures, as OPCAB patients were discharged promptly to cardiac rehabilitation clinics, consistent with the standard 4-day hospital stay for HCR patients. Notably, our HCR protocol involves a two-staged procedure with PCI performed at least 3 days after surgery. This conservative approach, which ensures effective stenting and minimizes bleeding risks by starting dual antiplatelet therapy on the third day, contributed to a longer hospital stay. In case PCI would be performed earlier, HCR might lead to a shorter postoperative stay.

In our HCR group, there were no initial conversions to sternotomy. However, within the first 30 postoperative days, MI occurred in 3.4% of the patients in the HCR group, which was more than in the OPCAB group (1.6%). Most of these MIs were related to issues with the LAD and were treated with stenting by cardiologists. These events may be attributed to initial surgeon inexperience, leading to technical flaws in graft anastomoses as well as suboptimal patient selection, such as patients with a gracile LAD or challenging anatomy. However, these incidents highlight the more challenging surgical procedure, further emphasized by the presence of 3 distinct learning curves. Each center started performing HCR at different times, and all patients, including the very first case of each program, were included, thereby reflecting varying levels of experience and adding complexity to the study. In the OPCAB group, 7 patients (2.2%) required repeat revascularization: 4 following MI and 3 due to graft failure identified by coronary angiography after abnormal ECG findings. These latter cases reflect strict postoperative monitoring, in which early detection and intervention likely prevented progression to MI. This explains the relatively higher rate of reported revascularization in OPCAB despite a lower incidence of MI.

Regarding mortality, at a median follow-up of 36 months, rates were 6.8% in the HCR group and 5.1% in the OPCAB group. Although numerically higher for HCR, this difference was not statistically significant (OR = 1.63, 95% CI: 0.58 to 2.80, *P* = 0.43). Most deaths in both groups were noncardiac, primarily oncologic or neurologic, indicating that midterm mortality reflected the comorbidity burden rather than the surgical strategy itself. At midterm follow-up, the need for TVR was observed in both the HCR and OPCAB groups, with rates of 9.4% and 6.1%, respectively. However, this difference was not statistically significant. These percentages, which include repeat revascularization within the first 30 days, were similar to the findings of Hage et al.^13^ However, when considering only TVR events occurring after patient discharge, these percentages decreased to 5.1% for HCR and 4.1% for OPCAB, respectively. This finding is in contrast with a meta-analysis by Harskamp et al.^14^ The latter study showed a higher repeat revascularization rate at 3 years for HCR (8.3%) compared with CABG (3.4%). In our population, the TVR rate for HCR was almost half of that noted in the Harskamp et al. study, likely attributable to the improved performance of modern stents. Our OPCAB outcomes align with the meta-analysis by Gaudino et al., which found no significant difference in the occurrence of late repeated revascularization between OPCAB and on-pump CABG.^5^ This suggests that our hospital’s surgical expertise in OPCAB contributes to minimizing unfavorable outcomes, thereby creating a robust control group for HCR. Long-term follow-up is needed to further evaluate the outcomes of these procedures.

HCR offers distinct advantages over OPCAB. The minimally invasive, sternal-sparing approach is highly valued by both patients and cardiologists, and it may support Enhanced Recovery After Surgery programs, potentially leading to shorter postoperative hospital stays.^15^ In addition, HCR avoids surgical maneuvers on the aorta and substitutes the most challenging grafting on the lateral and inferior walls with PCI.^15,16^ This could position HCR to gain prominence and possibly replace OPCAB.

Nevertheless, the future of HCR is uncertain; it might either persist as a specialized procedure akin to OPCAB, constrained by its inherent technical complexity, or be seen as an advancement over OPCAB, becoming more widely accepted for its minimally invasive nature. For HCR to transition into broader use, further research and validation are crucial, especially for the suitability of HCR in high-risk patients.

### Limitations

We acknowledge several limitations in our study. First, we relied on a prospective, observational dataset, which may introduce selection bias. Although we partially addressed this issue with PSM analyses, some bias may persist. Second, the HCR dataset comprised data from a national database, encompassing the learning curves of 3 centers, whereas the OPCAB dataset was from a single institution, potentially limiting generalizability. In addition, the absence of detailed coronary angiographic data, including SYNTAX scores and lesion location, precluded a more nuanced anatomical comparison between groups. Finally, the relatively low number of HCR cases and the sharp decline in patients at risk after 3 years limits the interpretation of midterm outcomes. Therefore, it is conceivable that the absence of statistically significant differences reflects insufficient follow-up duration and sample size rather than true equivalence between groups.

## Conclusions

Our study showed that in The Netherlands, HCR with RA-MIDCAB demonstrated comparable outcomes with OPCAB for MACCE at short-term follow-up as well as for TVR and survival at midterm follow-up in appropriately selected patients. However, given the relatively small number of HCR patients, the midterm results should be interpreted with caution. Further research with larger cohorts and extended follow-up is essential to thoroughly assess long-term outcomes.

## Supplemental Material

sj-docx-1-inv-10.1177_15569845261437076 – Supplemental material for Nationwide Short-Term and Midterm Clinical Outcome Comparison of Hybrid Coronary Revascularization Versus Off-Pump Coronary Artery Bypass Surgery Supplemental material, sj-docx-1-inv-10.1177_15569845261437076 for Nationwide Short-Term and Midterm Clinical Outcome Comparison of Hybrid Coronary Revascularization Versus Off-Pump Coronary Artery Bypass Surgery by Monica Gianoli, Anne R. de Jong, Ferenc van der Hulst, Maaike M. Roefs, Sandeep Singh, Patrique Segers, Pim van der Harst, Willem J.L. Suyker and Kirolos A. Jacob in Innovations
