# Supplementary material for: Nationwide Short-Term and Midterm Clinical Outcome Comparison of Hybrid Coronary Revascularization Versus Off-Pump Coronary Artery Bypass Surgery
Source: Innovations (Phila). 2026 Apr 23;21(2):150–8. doi: 10.1177/15569845261437076 (PMC13260735; doi:10.1177/15569845261437076)
Supplement: sj-docx-1-inv-10.1177_15569845261437076 – Supplemental material for Nationwide Short-Term and Midterm Clinical Outcome Comparison of Hybrid Coronary Revascularization Versus Off-Pump Coronary Artery Bypass Surgery [file sj-docx-1-inv-10.1177_15569845261437076.docx]

**Supplemental Material**

**Appendix 1.** List of Members of the Netherlands Heart Registration Commission.

***Cardiothoracic Surgery***

Dr. S. Bramer - Amphia Ziekenhuis

Dr. R.A.F. de Lind van Wijngaarden - Amsterdam UMC

Dhr. B.M.J.A. Koene - Catharina Ziekenhuis

Dr. J.A. Bekkers - Erasmus MC

Dr. G.J.F. Hoohenkerk - HagaZiekenhuis

Dr. A.L.P. Markou - Isala

Dhr. A. de Weger - Leids Universitair Medisch Centrum

Dr. P. Segers - Maastricht UMC+

Dr. D. Stecher - Medisch Centrum Leeuwarden

Dr. R.G.H. Speekenbrink - Medisch Spectrum Twente

Dr. V.G. Hindori - Onze Lieve Vrouwe Gasthuis

Dhr. W.W.L. Li - Radboudumc

Dhr. E.J. Daeter - St. Antonius Ziekenhuis

Dr. M.M. Mokhles - UMC Utrecht

Dr. Y. Douglas - Universitair Medisch Centrum Groningen

***Interventional Cardiology***

Dr. J.M. Cheng - Albert Schweitzer ziekenhuis

Dr. M. Meuwissen - Amphia Ziekenhuis

Prof. dr. J.P.S. Henriques - Amsterdam UMC, locatie AMC

Dr. K.M.J. Marques - Amsterdam UMC, locatie VUmc

Dr. R. Al Hashimi - Canisius Wilhelmina Ziekenhuis

Dr. K. Teeuwen - Catharina Ziekenhuis

Dr. M. Magro - Elisabeth-TweeStedenZiekenhuis

Dr. R. Diletti - Erasmus Medisch Centrum

Dhr. B.J. Sorgdrager - Haaglanden Medisch Centrum

Dhr. C.E. Schotborgh - HagaZiekenhuis

Dr. R.J.R. Snijder - Isala

Dr. J. Polad - Jeroen Bosch Ziekenhuis

Dr. R. Scherptong - Leids Universitair Medisch Centrum

Dr. M. van der Ent - Maasstad Ziekenhuis

Prof. dr. A.J.W. van 't Hof - Maastricht UMC+, Zuyderland Medisch Centrum

Dhr. F. Spano - Meander Medisch Centrum

Dhr. J. Brouwer - Medisch Centrum Leeuwarden

Dhr. K.G. van Houwelingen - Medisch Spectrum Twente

Dr. A. Dedic - Noordwest Ziekenhuisgroep

Dr. G. Amoroso - Onze Lieve Vrouwe Gasthuis

Dhr. C. Camaro - Radboudumc

Dr. P.W. Danse - Rijnstate

Dr. K. Sjauw - St. Antonius Ziekenhuis

Dr. E.K. Arkenbout - Tergooi

Dhr. W.T. Ruifrok - Treant Zorggroep

Dr. A.O. Kraaijeveld - UMC Utrecht

Dr. E. Lipsic - Universitair Medisch Centrum Groningen

Dhr. S. Aydin - Viecuri Medisch Centrum

Dhr. R. Erdem - ZorgSaam Ziekenhuis

**Appendix 2.** Surgical Techniques and Percutaneous Coronary Intervention.

***Surgical Techniques***

The procedural details for robot-assisted minimally invasive direct coronary artery bypass have been outlined in detail by our team.^1^ In brief, the patient underwent general anesthesia and was positioned at a 30-degree right lateral decubitus. Using the da Vinci system by Intuitive Surgical (Sunnyvale, CA, USA), the left internal thoracic artery (LITA) was harvested. Following LITA harvesting, the pericardium was opened to expose the left anterior descending artery (LAD). Once appropriate heparinization was achieved (if the activated clotting time >300 seconds), the LITA was ligated. Subsequently, either the endoscopic Octopus Nuvo (Medtronic, Dublin, Ireland) or Acrobat-i Stabilizer (Getinge Group, Göteborg, Sweden) off-pump stabilizer was introduced, either through a prior port site or directly through the minithoracotomy, to stabilize the LAD. The LITA-to-LAD conduit was manually anastomosed using off-pump beating cardiac techniques.

The off-pump coronary artery bypass (OPCAB) procedure was performed at the University Medical Center Utrecht by 2 surgeons. Following a median sternotomy, a deep stitch was placed on the posterior side of the pericardium to facilitate heart manipulation. The Acrobat-i Stabilizer was used to stabilize the anastomotic site. Proximal anastomoses were typically carried out with partial aortic clamping, although the Heartstring® device (Getinge Group) was alternatively employed. In addition, nonaortic grafting techniques were employed, using the LITA in situ combined with composite grafts. The cell-saving system was routinely employed during the OPCAB procedure. In both surgical techniques, intracoronary ClearView™ (Medtronic) shunts were used to maintain distal perfusion during coronary anastomosis, where applicable.

***Percutaneous Coronary Intervention (PCI)***

The PCI has been documented in prior work by our team.^1^ PCI involving stenting of non-LAD vessels was conducted in a two-staged manner by an interventional cardiologist, employing established techniques. Patients were implanted with second-generation drug-eluting stents and received a combination of lifelong aspirin alongside ticagrelor or clopidogrel for up to 12 months, adjusted as per individual bleeding risk assessments. In reverse hybrid coronary revascularization cases, dual antiplatelet therapy was maintained; however, ticagrelor was switched to clopidogrel 5 days prior to surgery.

**Reference**

1. de Jong AR, Gianoli M, Namba HF, et al. A nationwide study of clinical outcomes after robot-assisted coronary artery bypass surgery and hybrid revascularization in the Netherlands. *Innovations* 2023; 18: 73–79.

**Supplemental Table 1.** Operative characteristics after propensity score matching for patients who underwent HCR compared with patients who underwent OPCAB between 2015 and 2021 in the Netherlands.

| **Operative and interventional characteristics** | **HCR study population (*n* = 117)** | **OPCAB study population (*n* = 313)** |
| --- | --- | --- |
| LIMA-LAD | 117 (100) | 313 (100) |
| Bypass per patient | 1.0 ± 0 | 3.6 ± 1.1 |
| Bypass type |  |  |
| LIMA >1 | 0 | 131 (41.8) |
| RIMA | 0 | 32 (10.2) |
| Radial artery | 0 | 30 (9.6) |
| VSM | 0 | 243 (77.6) |
| Urgent surgical revascularization | 20 (17) | 161 (51.4) |
| Conversion to sternotomy | 0 | — |
| HLM use | 0 | 0 |
| Multivessel disease |  |  |
| Two vessels | 93 (79.5) | 112 (35.8) |
| Three vessels | 24 (20.5) | 201 (64.2) |
| HCR type |  | — |
| Two-staged procedure | 117 (100) |  |
| Planned procedure | 56 (47.9) |  |
| Reversed two-staged procedure | 61 (52.1) |  |
| HCR procedure |  | — |
| DES | 105 (91.3) |  |
| Balloon dilation | 6 (5.1) |  |
| Unknown | 6 (5.1) |  |
| *Coronary targets* |  |  |
| Total stents of bypasses | 139 (100) | 1,131 (100) |
| Stent or bypass position |  |  |
| LAD | 0 | 513 (45.3) |
| CX | 56 (40.3) | 302 (26.7) |
| IM | 3 (2.1) | 68 (6.0) |
| RCA | 79 (56.1) | 248 (21.9) |
| Unknown | 1 (0.7) | 0 |

Abbreviations: CX, circumflex coronary artery; DES, drug-eluting stent; HCR, hybrid coronary revascularization; HLM, heart-lung machine; IM, ramus intermedius artery; LAD, left anterior descending artery; LIMA, left internal mammary artery; OPCAB, off-pump coronary artery bypass; PCI, percutaneous coronary intervention; RCA, right coronary artery; RIMA, right internal mammary artery; VSM, vena saphena magna.

Data are reported as counts (%) or mean ± standard deviation, unless otherwise indicated.

**Supplemental Figure 1.** Distribution of estimated propensity scores prior to matching in patients undergoing HCR or OPCAB. The histogram demonstrates good overlap between treatment groups and supports the assumption of common support required for propensity score matching.


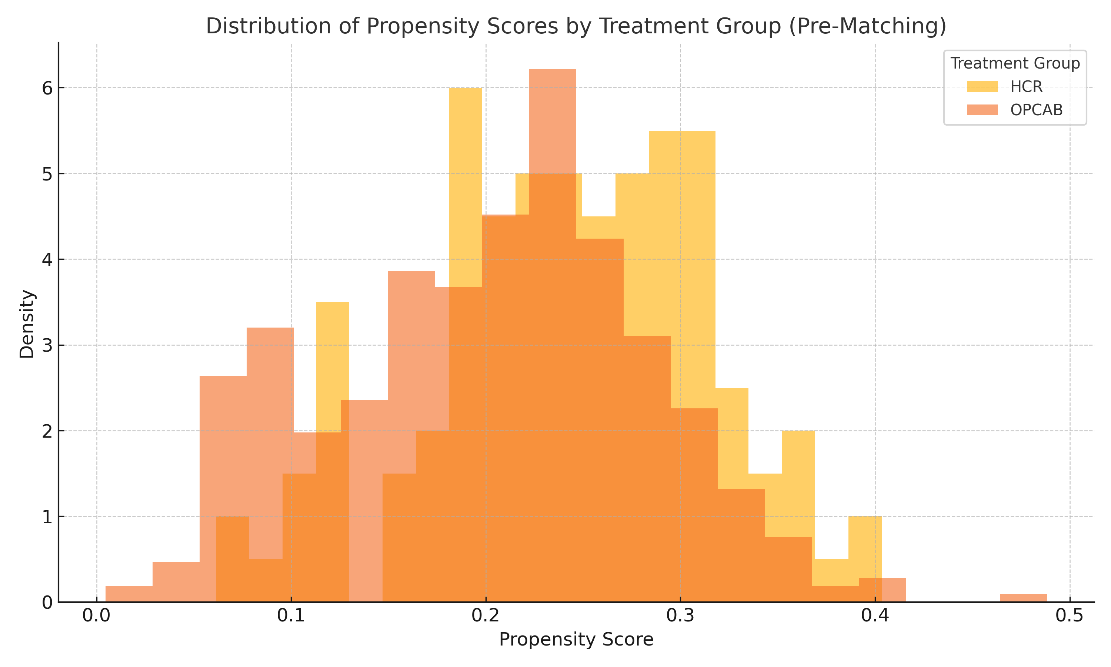


Abbreviations: HCR, hybrid coronary revascularization; OPCAB, off-pump coronary artery bypass.

**Supplemental Figure 2.** Distribution of propensity scores after matching in the HCR and OPCAB groups. The overlap indicates successful matching and confirms balance between the treatment arms.


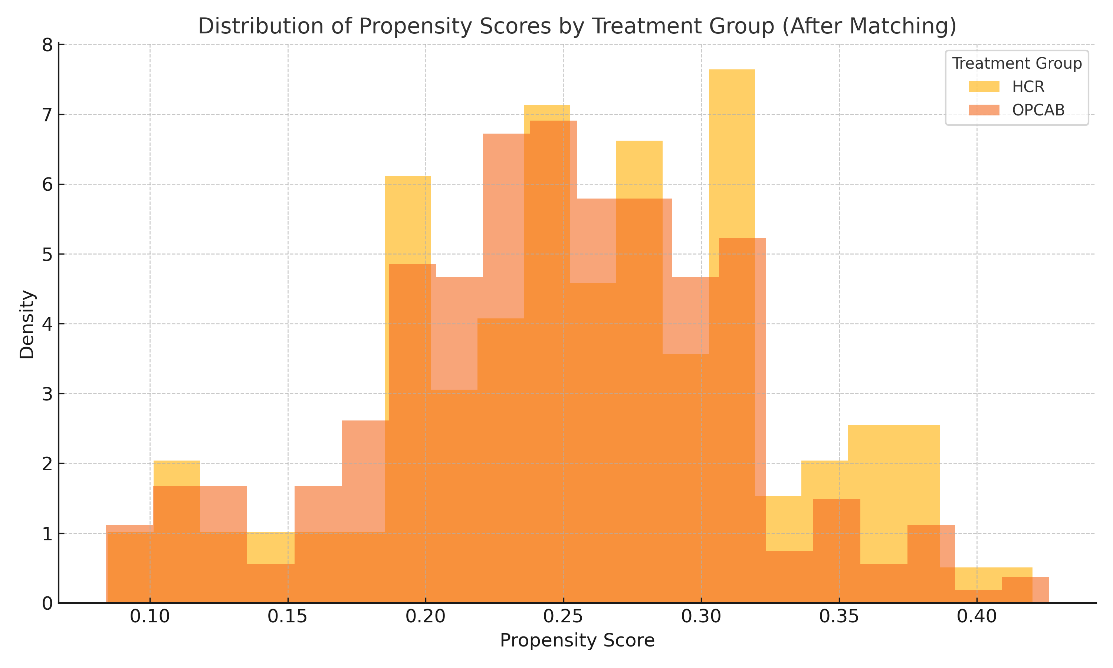


Abbreviations: HCR, hybrid coronary revascularization; OPCAB, off-pump coronary artery bypass.
